# AlphaFold 3: an unprecedent opportunity for fundamental research and drug development

**DOI:** 10.1093/pcmedi/pbaf015

**Published:** 2025-07-01

**Authors:** Ziqi Fang, Hongbiao Ran, YongHan Zhang, Chensong Chen, Ping Lin, Xiang Zhang, Min Wu

**Affiliations:** School of Medical Information Engineering, Gannan Medical University, Ganzhou 341000, China; RNA Research and Drug Discovery Center, Wenzhou Institute, University of Chinese Academy of Sciences, Wenzhou 325000, China; RNA Research and Drug Discovery Center, Wenzhou Institute, University of Chinese Academy of Sciences, Wenzhou 325000, China; Integrative Science Center of Germplasm Creation in Western China (Chongqing) Science City, Biological Science Research Center, Southwest University, Chongqing 400715, China; RNA Research and Drug Discovery Center, Wenzhou Institute, University of Chinese Academy of Sciences, Wenzhou 325000, China; Department of Graduate College, Zhejiang Chinese Medical University, Hangzhou 310000, China; The Joint Research Center, Affiliated Xiangshan Hospital of Wenzhou Medical University, Ningbo 315000, China; Integrative Science Center of Germplasm Creation in Western China (Chongqing) Science City, Biological Science Research Center, Southwest University, Chongqing 400715, China; School of Medical Information Engineering, Gannan Medical University, Ganzhou 341000, China; School of Medical Information Engineering, Gannan Medical University, Ganzhou 341000, China; RNA Research and Drug Discovery Center, Wenzhou Institute, University of Chinese Academy of Sciences, Wenzhou 325000, China; The Joint Research Center, Affiliated Xiangshan Hospital of Wenzhou Medical University, Ningbo 315000, China; Department of Medicine, Harvard Medical School, and Brigham and Women's Hospital, Boston, MA 02115, USA

**Keywords:** AlphaFold3, artificial intelligence, structure prediction, drug design, biomedical research

## Abstract

AlphaFold3 (AF3), as the latest generation of artificial intelligence model jointly developed by Google DeepMind and Isomorphic Labs, has been widely heralded in the scientific research community since its launch. With unprecedented accuracy, the AF3 model may successfully predict the structure and interactions of virtually all biomolecules, including proteins, ligands, nucleic acids, ions, etc. By accurately simulating the structural information and interactions of biomacromolecules, it has shown great potential in many aspects of structural prediction, mechanism research, drug design, protein engineering, vaccine development, and precision therapy. In order to further understand the characteristics of AF3 and accelerate its promotion, this article sets out to address the development process, working principle, and application in drugs and biomedicine, especially focusing on the intricate differences and some potential pitfalls compared to other deep learning models. We explain how a structure-prediction tool can impact many research fields, and in particular revolutionize the strategies for designing of effective next generation vaccines and chemical and biological drugs.

## Graphical Abstract

**Graphical Abstract:**
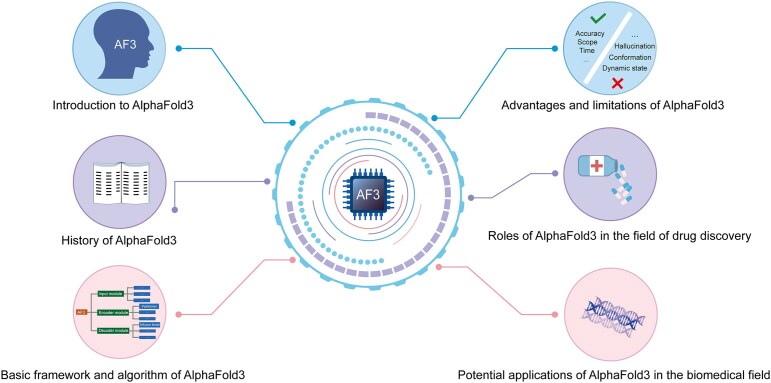
Overview, application, and perspective of AlphaFold3.

## Introduction

The 2024 Nobel Prize in Chemistry was awarded to Demis Hassabis and John Jumper of the DeepMind team in recognition of their breakthrough contributions to the development of the AlphaFold series [1]. This honor not only highlights the revolutionary role of artificial intelligence (AI) in the field of protein structure prediction, but also marks the advent of a new era of computational biology. It is notable that several months before the awarding of this prize, DeepMind, in collaboration with Isomorphic Labs, published its latest research result based on AlphaFold3 (AF3) in the journal *Nature* [2]. AF3, while retaining its original technological advantages, immensely expands its prediction range from a single protein to almost all biomolecular systems. By introducing an innovative diffusion model architecture, AF3 has powerfully improved prediction accuracy. This leapfrog development enables algorithm-driven structural prediction to no longer be limited to a single molecular type, opening up brand-new research directions and application prospects for the entire field of structural biology [3, 4]. Although AF3 itself still has certain flaws, it is undeniable that its emergence is expected to effectively address the current challenges in biophysical structure analysis and molecular interaction prediction, infusing new vital nutrients and energy into several important fields, such as biomedical research, drug design, and disease diagnosis and treatment [5].

Despite the short period since its birth, discussions about the value of AF3 have never ceased. To gain a deeper understanding of the potential and challenges of AF3 in disease treatment, this article provides an overview of AF3’s development, working principles, and application prospects. By sorting out the innovative applications of AF3 in drug discovery and biomedical research, and objectively analyzing its technical limitations, the aim of this article is to provide comprehensive analyses and inspiration for scientific researchers in broad fields.

## History of AlphaFold predicting protein structure

**Figure 1.**
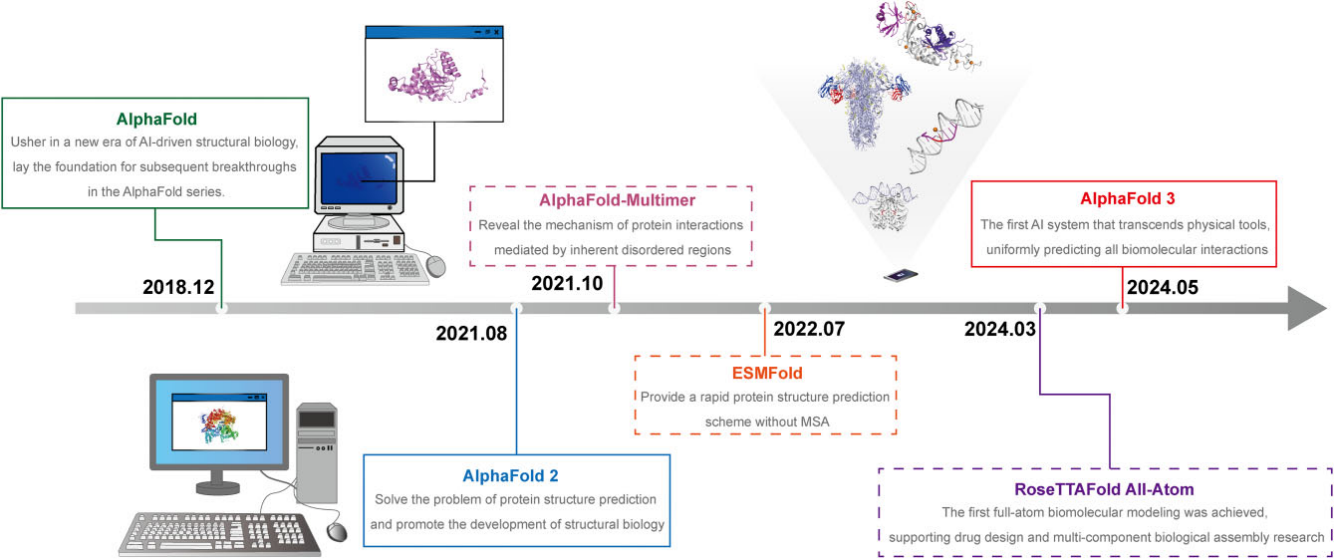
Development history and key breakthroughs timeline of the AlphaFold series of models. The diagram showcases the milestone progress from the first-generation AlphaFold initiating a new era of AI-driven structural biology in 2018 to AlphaFold 3 achieving full prediction of biomolecular interactions in 2024. In addition to the AlphaFold family, this figure also describes its extension algorithms and other protein prediction models. The core contributions of each model are as follows: AlphaFold 2 solves the problem of protein structure prediction (2021), AlphaFold-Multimer reveals the mechanism of disordered protein interactions (2021), and ESMFold achieves rapid prediction without multiple sequence alignment (MSA) (2022). RoseTTAFold All-Atom completed the full-atom biological modeling (2024). The time nodes mark the release time of each model, reflecting the technological update trajectory in this field.

As the cornerstone of life activities and the core regulatory executive, protein is widely involved in and profoundly affects almost all life processes in the cell. The universality and diversity of its functions, fundamentally speaking, come from the unique 3D spatial structure of protein molecules [6]. Therefore, accurate prediction of protein structure is of great significance for deepening our understanding of life phenomena, revealing the molecular mechanisms of disease occurrence, and drastically accelerating the process of drug discovery. Since 1957, when John C. Kendrew and Max F. Perutz first revealed the 3D structure of proteins through X-ray crystallography [7], the ambition for resolving protein structure has never stopped [8–13]. Traditional prediction models face a number of challenges, such as requiring an available experimental database, being time-consuming, requiring a large number of calculations, and having a narrow prediction range, hence, the large-scale popularization of protein structure analysis has been severely hampered [14–16]. Fortunately, the rise of AI technology has brought new opportunities for development in this field. With its high precision and high efficiency, the structure prediction model has been sought after by the majority of biological structure scholars since its emergence [17–19]. Among them, the AlphaFold series models have become a prominent leader in the field of protein structure prediction thanks to their excellent performance (Fig. 1).

### AlphaFold

In 2018, DeepMind, which rose to fame due to AlphaGo [20], introduced an algorithm model, now well-known as AlphaFold, and its release immediately attracted great academic attention [21]. To improve prediction accuracy, AlphaFold uses an advanced generative neural network architecture and is optimized with gradient descent algorithms. With this technique, researchers are able to predict the distances between pairs of amino acids and the angles between the chemical bonds that connect these amino acids. This information has important guiding ability and is extremely critical for improving mechanistic research and disease treatment. In that year's International Critical Assessment of Structure Prediction (CASP) competition, AlphaFold won first place with its excellent performance, further showing its leading position in the field of protein structure prediction [22, 23]. Unfortunately, AlphaFold still has deficiencies in prediction accuracy and is limited by its own modularity when dealing with local protein interactions as well as broader structural features. This affects AlphaFold's effectiveness in some specific application scenarios, making it relatively limited in scope.

### AlphaFold2

AlphaFold2 (AF2) was officially launched in 2021 [24]. But before that, it had already won the championship of the 14th CASP [25]. During the competition, this program demonstrated a predictive ability far exceeding that of other contestants, and its results were nearly consistent with the structures observed by cryo-electron microscopy [10]. By leveraging its high-precision predictive capabilities, AF2 successfully predicted >200 million protein structures [26] and topped the list of Science's annual scientific Breakthroughs in 2021 for its contribution [27]. Since its launch, AF2 has been widely used in drug development [28], biological mechanism research [29–38], and protein function prediction [39–43], becoming one of the indispensable tools in the field of structural biology. Researchers have developed several new models [such as AlphaFold Multitimer (AFM) [44], PCMol [45], etc.], which play important roles in the fields of reproductive biology [46] and drug design. Although AF2 currently remains active, it has certain limitations, including the accuracy of protein prediction, the high hardware requirements, the uniformity of protein conformation prediction, and the oneness of the prediction object [47]. These shortcomings mean that experimental structural biology still plays a leading role to some extent, and protein prediction through AI still needs further innovation and development.

### AlphaFold3

To address the inherent defects, DeepMind continued refining its program and released AF3 on 8 May 2024, marking a major breakthrough in AI in the field of biological science, especially in the prediction of biomolecular structure. As the latest version of the AlphaFold series, AF3 not only inherits the outstanding performance of its predecessor AF2 in protein structure prediction, but also significantly expands its application scope through technological innovation, enabling it to accurately predict the complex structure of small molecules, nucleic acids, substrates, and other ligands. These features make AF3 stand out among the various existing deep learning tools, opening up a broader prospect for research in the field of biological sciences (Table 1). However, as with all emerging technologies, AF3 is not perfect and still has shortcomings in the prediction of structural conformation and dynamic behavior. These problems may be solved with the open-source optimization of AF3 [48]. In conclusion, the emergence of AF3 has had a profound impact on the field of structural biology, greatly expanding the scope of basic science research, facilitating the development of structure analysis of biological macromolecules and the prediction of molecular interaction.

**Table 1.** Comparison of the five mainstream protein structure prediction tools.

Trait	AF3	AF2	AlphaFold-Multimer	RoseTTAFold All-Atom	ESMFold
Release time	May 2024	August 2021	October 2021	March 2024	July 2022
Main objective	Predict almost all biomolecular complexes	Predict protein monomers and some complexes	Predict the structure of protein–protein complexes	Predict the structure of all-atomic biomolecular complexes	Rapid prediction of protein monomers
Input data	Multiple types of biomolecular sequences	Standard amino acid sequence	Multi-protein chain sequence	Amino acid/nucleic acid sequence	Only protein sequences (without multiple sequence alignment)
Output result	The 3D structure and interaction of proteins and their complexes	3D structure of protein	Protein–protein complex structure	The structure of all-atomic biomolecular complexes	3D structure of protein
Algorithm optimization	The pairformer model and the diffusion model	Based on transformers and evolution models	Based on AF2, it supports multi-chain prediction and symmetric processing.	All-atomic modeling	Pure language model
Application scope	Prediction of structures and interactions of proteins, nucleic acids, small molecule ligands, ions, covalent modifications, etc.	Single protein structure prediction	Research on the structure and mechanism of action of protein–protein complexes	Protein–small molecule docking and covalent modification	Large-scale high-throughput protein structure prediction
Accuracy	Compared with AF2, the accuracy has been further improved and it outperforms professional tools in the vast majority of structural predictions.	CASP14 has reached a level close to experimental accuracy.	It performs well in predicting the interactions between proteins (including disordered structures).	It is superior to the traditional docking method in the prediction of protein–small molecule complexes but inferior to AF3.	It is close to the accuracy of AF2, but the prediction of some complex structures is slightly inferior.
Open source situation	Open source	Open source	Not officially open-sourced	Open source	Open source

## Working principle of AF3

**Figure 2.**
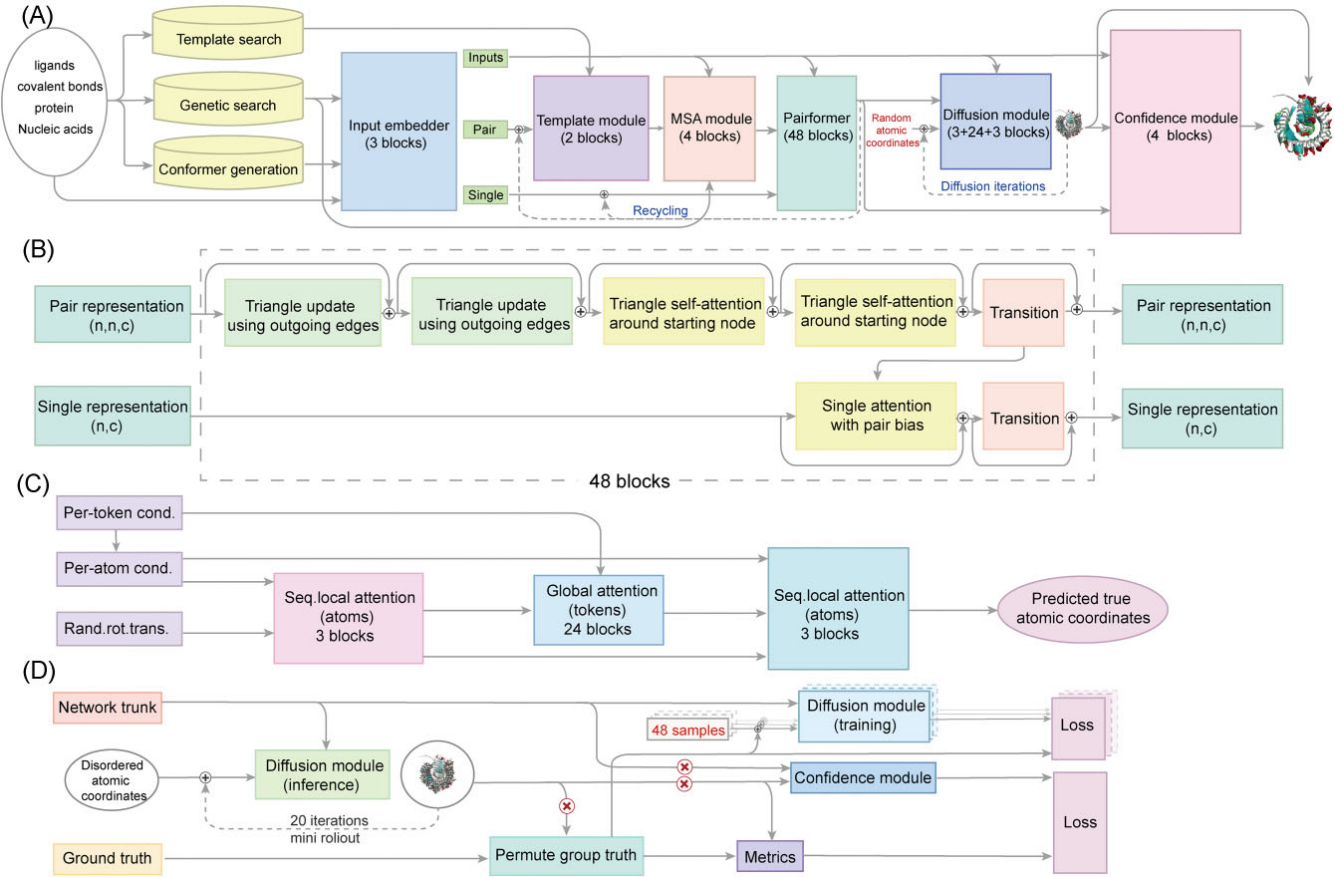
Basic framework of AF2 and working principle diagram of each component. (**A**) Overall framework diagram of AF3. The overall framework of AF3 is divided into three main parts. The first part is the input module, which is responsible for collecting and integrating MSA and template structure information as input data for the entire model. The second part is the encoder module, which adopts a pairformer module to replace the evoformer module in AF2 to achieve the processing of input data and feature extraction. The third part is the decoder module, the core of which is the diffusion module. It can generate the 3D structure of target biological macromolecules by denoising. (**B**) Working principle diagram of the pairformer module. This module takes the pair representation and single representation output of the previous module as input data, and only keeps four MSA sequences, which greatly simplifies the process and shortens the processing time. (**C**) Diffusion module, as a mini rollout. Using raw atomic coordinates and coarse-grained abstract token representations as input objects, the predicted atomic coordinates are generated directly without twisting or equivariable processing. (**D**) Diffusion training diagram. The module is able to handle the training of 48 samples at the same time, and the error is predicted by the confidence module.

As a highly regarded deep learning model for protein structure prediction, the core concept of AF3 is rooted in the deep integration of deep learning and protein amino acid sequence characteristics. This model achieves high-precision prediction of the 3D structure of proteins by integrating the advantages of convolutional neural networks and residual networks and combining with large-scale data training. In terms of architectural design, AF3 continues the core framework of AF2 and still adopts multiple sequence alignment (MSA) as the main input feature. However, in order to achieve a better prediction effect, AF3 has made significant adjustments on this basis (Fig. 2A). The new architecture introduces a more concise Pairformer [49] to replace the Evoformer module in AF2, reducing the processing burden of MSA, and focuses more on extracting critical evolutionary information. In order to simplify the machine learning architecture and improve the prediction efficiency, AF3 uses the diffusion model to replace the traditional structure generation module. This reduces the complexity of the model and the loss of stereochemical information. Similarly, by eliminating the global rotational and translational invariance or equivariance of the molecules, AF3 further optimizes the computational process, enabling the model to focus primarily on the prediction of core structural features.

The detailed architecture of AF3 has been fully elaborated in the original article [2]. For the convenience of the journal's diverse readership, we briefly outline its architecture and rationale. Similar to AF2, AF3’s model framework is made up of three parts.

### Input module

The input module of AF3, as the primary link of the entire prediction process, undertakes the key tasks of data integration and feature extraction. Its core responsibility is to construct a comprehensive initial dataset by integrating evolutionary information (MSA) and known structural templates. Compared to AF2, the key breakthrough of the AF3 input module lies in the introduction of the conformer generation technology, which can automatically generate the initial 3D structure based on intramolecular interaction law, providing a more reasonable starting point for subsequent predictions.

In the input module of AF3, the raw input data covers a wide range of biomolecular information, including but not limited to amino acid sequences, ligands, covalent bonds, etc. To better handle this information, AF3 adopts a hierarchical feature extraction mechanism. Specifically, for conventional amino acids and nucleotides, the system adopts an overall characterization approach. For modified residues and small-molecule ligands, atomic-level fine analysis enables the complete retention of molecular details. This differentiated processing approach should be taken into account for both computational efficiency and prediction accuracy. Subsequently, the system converts the molecular information into high-dimensional vector representations through a feature-embedding process. Among them, the pair representation is used to represent interactions between pairs of residues, revealing their spatial proximity and possible chemical bonding. Single representation focuses on capturing the characteristics of individual residues, including their chemical properties, shapes, and patterns of interaction with other residues. This transformation retains the biological characteristics of the original data, and establishes the correlation network among tokens through the attention mechanism, laying a solid foundation for AF3’s protein structure prediction task.

### Encoder module

AF3’s encoder module adopts a three-stage feature extraction architecture, including the Template module, the MSA module, and the Pairformer module, which jointly achieve the deep processing and feature extraction of input data.

In the initial stage, template structures acquire known structural templates similar to the target sequence through a structural homology search, and encode the spatial conformational features of these templates into computable mathematical representations. In essence, this process utilizes the rich known structural information in the structural database to assist in predicting the unknown protein structure, thus improving the accuracy and reliability of the prediction.

On this basis, the developers further use the MSA module [50] to process information and identify the co-evolutionary signals among residues from large-scale sequence alignment. These signals reveal the spatial and functional interactions formed by amino acids under evolutionary pressure, providing key constraints for inferring 3D spatial proximity.

The Pairformer module [49] is the core component of the encoder module, mainly responsible for resolving the local interactions between adjacent residues. This module adopts an improved transformer architecture. Based on inheriting the 48-layer stacked design of the Evoformer module in AF2, it retains key algorithm components, such as triangular update and triangular self-attention (Fig. 2B). On this basis, this model significantly improves the efficiency of feature extraction through architectural innovation. It simplifies the multi-sequence alignment processing to four sequences, significantly reducing the computational complexity. Meanwhile, the expression methods centered on residue pairs (preliminary single representation and pair representation) are adopted to capture the spatial relationship between adjacent residues more accurately. This design significantly reduces the model's reliance on evolutionary information while maintaining high-precision structural prediction capabilities.

In the course of Pairformer's work, there is a complex interaction between single representation and pair representation. Although a single representation does not directly affect the expression of a pair representation, a pair representation can influence the flow of information in a single representation through the single attention with pair bias mechanism. This interaction enables the encoder module to capture and integrate the information in the input data more comprehensively, providing a richer and more accurate feature representation for the subsequent decoding process.

After multiple layers of repeated optimization, the encoder finally outputs high-quality single representation and pair representation. These two representations are fed into the decoder module to further generate and optimize the 3D structure of the protein.

### Decoder module

Compared to AF2, AF3’s decoder module has undergone significant innovation. Its core is the introduction of diffusion-based generative modeling [49] to replace the iterative optimization framework of AF2. Its working principle is to directly predict the atomic coordinates through the reverse process of multiple rounds of noise addition and removal (Fig. 2C), achieving a progressive construction from noise distribution to fine structure. This innovative mechanism enables the model to gradually correct the predictions, so that, in the early denoising stage, large-scale noise is processed to determine the overall conformational framework, and in the subsequent denoising stage, the fine adjustment of the local structure is focused on, ultimately generating accurate atomic positions. Compared to the direct structure prediction by AF2, this generation strategy acts on the original atomic coordinates and coarse-grained abstract token representations, reducing labor costs. Moreover, it can integrate multi-scale features through noise scheduling, handle complex ligand environments more naturally, and avoid conformational constraints introduced by torsional angle parameterization. Its parallel training architecture supports the simultaneous processing of 48 samples. These creative steps significantly improve the training efficiency and model generalization ability.

The diffusion module adopts a dual-mode design, including the inference diffusion module and the training diffusion module (Fig. 2D). The inference diffusion module primarily serves as the test phase. The diffusion model starts from random noise and generates accurate atomic coordinates through multi-round denoising combined with the attention mechanism. The training diffusion module is responsible for the training phase. It takes a known protein structure model from the database [usually derived from Protein Database (PDB) and MSA], superimposes controllable Gaussian noise on the real structure, and then trains the diffusion model to restore the real coordinates. This multi-scale training strategy enables the model to focus on the precise modeling of local chemical bonds under low-noise conditions and emphasizes the feature learning of the overall conformation in a high-noise environment, achieving collaborative structural prediction from micro to macro.

The generative diffusion model brings many advantages to AF3 and can overcome the limitations inherent in AF2 to some extent. However, its existence has also caused some new problems, the most prominent of which is the phenomenon of hallucination [51]. To counter this, AF3's developers introduced cross-distillation technology and used the prediction results of the more complex model (AFM v.2.3) as the supervisory signal to guide the model to correctly identify the disordered regions. In AF2, an unordered region is represented as a long-extended loop structure. By learning and analyzing this structure, AF3 is able to construct disordered regions more efficiently, thus mitigating hallucinatory effects. It is worth noting that cross-distillation does not simply copy the predictions of another model, but uses those results as references to train the current model. This method not only expands the diversity of the training data, but also effectively suppresses the generation of false structures by imitating the specific conformations of the disordered regions. To evaluate the quality of predictions, AF3 has established a comprehensive quality assessment system. By comparing the differences between the predicted structure and the experimental data at multiple scales (including local conformational accuracy, global topological matching degree, etc.), the system provides a credibility score for each prediction result. Generally speaking, the higher the confidence score, the higher the accuracy of the prediction result. These innovations enable AF3 to achieve more accurate and reliable prediction of biomolecular structures while maintaining efficient computing.

## Application potential of AF3 in drug design and biomedicine

**Figure 3.**
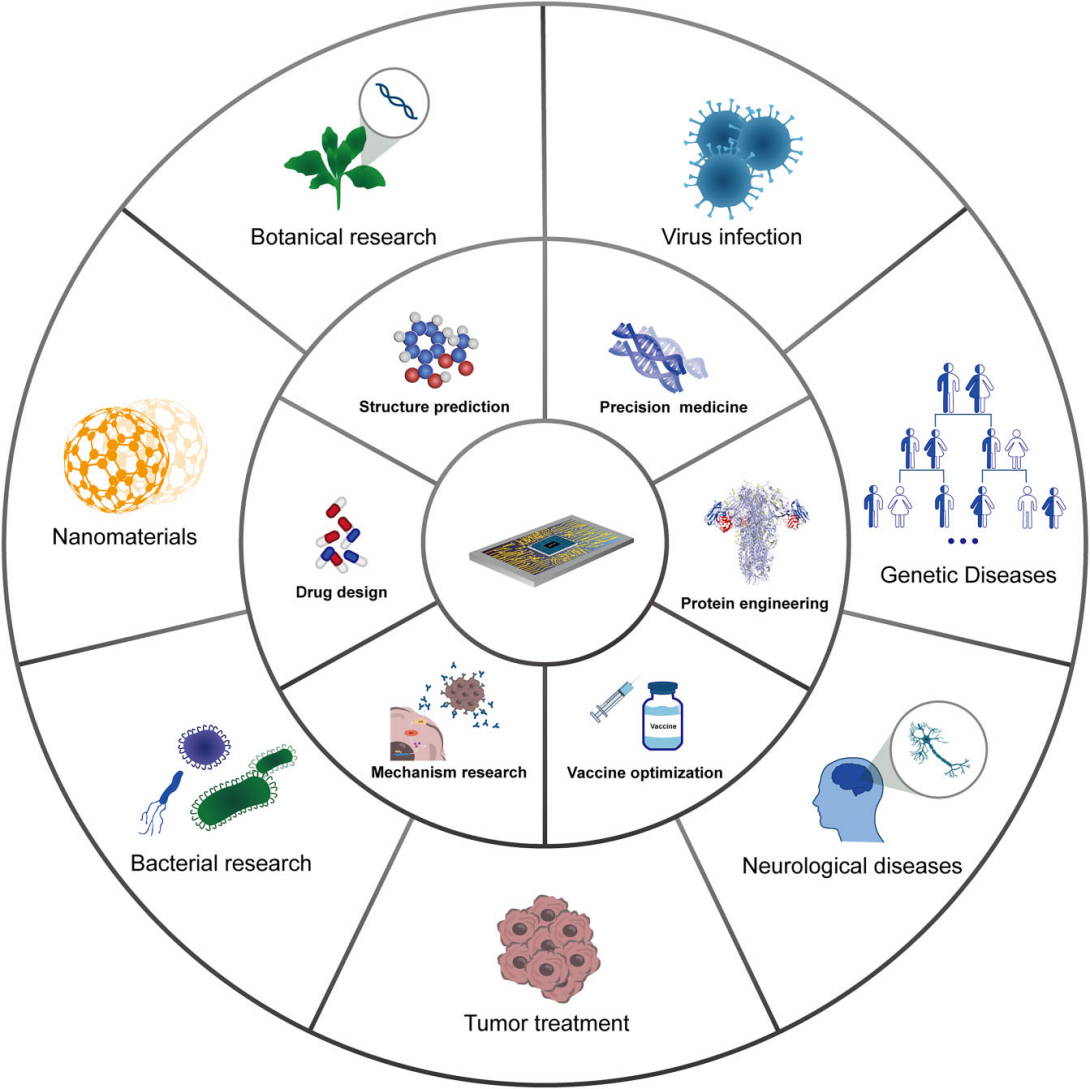
Multi-field applications of AF3. Because of its excellent performance, AF3 has broad application prospects in many fields. These fields include basic research (mechanism research, botanical research, virus infection, bacterial research), structural biology (structure prediction, protein engineering), medical applications (precision medicine, tumor treatment, neurological diseases, and genetic diseases), drug development (drug design, vaccine optimization, and nanomaterials) etc. The diagram highlights the potential extensive value of AF3 from molecular mechanism analysis to clinical transformation, demonstrating its multi-functionality as a core tool in computational biology.

In the wave of science and technology in the 21st century, research in the field of the life sciences has made substantial progress. Among such research, the development of drug discovery and biomedicine has benefited from the progressive understanding of the structure of biological macromolecules. Whether it is the understanding of the nature of life or the optimization of disease treatment, how to efficiently obtain accurate structural information of biological macromolecules is key to success. With the continuous development of AI, the prediction of a drug's molecular structure is no longer simply by means of classically standard experiments. More and more deep learning models have gradually appeared in the public eye, providing efficient and convenient prediction tools for structural biology, and promoting the rapid development of various fields [52]. In this context, the emergence of AF3 undoubtedly provides researchers with a new shortcut, allowing them to explore the micro world to a greater extent and use the results for the benefit of mankind. With its strong performance, AF3 may have great application potential in many disciplines and inject new energy and vitality into the development of the life sciences (Fig. 3).

### Drug design

Drug design has traditionally been regarded as a complex and time-consuming scientific task. The key lies in the in-depth understanding of the fine structure of molecular structure in living organisms and the intricate mechanism of molecular interaction. This understanding forms the logical starting point for drug development and strategies to treat disease. Traditional methods primarily rely on experimental techniques, such as X-ray crystallography and cryo-electron microscopy. Although these methods are precise, they are time-consuming and labor-intensive. The resolution of a single protein structure usually takes months or even years. This severely limits the efficiency of research and development. Although breakthroughs have been made in drug design based on the known structures of the PDB, it is still limited by data coverage and quality issues. In recent years, the vigorous development of AI technology has brought revolutionary changes to the field of drug research and development [53–58], among which the performance of the AlphaFold series of algorithms is particularly remarkable [59–63].

AF3, as the latest version of this series, has achieved significant breakthroughs in both prediction accuracy and application scope. Unlike the second generation, AF3 not only maintains the high accuracy of protein monomer structure prediction, but also extends the prediction range to complex systems including protein–ligand and protein–nucleic acid. In the PoseBusters benchmark test [64], the prediction accuracy of AF3 is much better than that of traditional molecular docking tools, and it does not require structural information. This marks that for the first time AF3 has surpassed physics-based AI systems in the field of biomolecular structure prediction. When compared to AFM and ColabFold [65], AF3 has also demonstrated excellent predictive ability for the structure of protein–peptide complexes [66]. These technological breakthroughs provide a brand-new methodological basis for drug design, enabling researchers to understand the mechanisms of molecular interactions at the atomic scale. With this advantage, AF3 successfully predicted >14 000 post-translational modification (PTM) protein–ligand complexes, opening up new avenues for exploring the interaction between PTM and drugs [67].

The test results of the SKEMPI database show that, compared to FoldX [68] and BindProfX [69], AF3 has unique advantages in estimating binding energy and free energy. In addition, combining it with deep learning methods like force fields and profilers can significantly improve the prediction performance [70]. Therefore, this model has been widely applied in the detection of various binding abilities, including protein–protein [71], protein–ligand [72] etc. These verifications and applications have laid a solid theoretical and practical foundation for its wide application in drug development and other related fields.

In terms of target identification, AF3 also performs well, and is especially suitable for the research and development of targeted drugs. The design principle of this type of drug is mainly based on antigen–antibody specificity or peptide affinity to achieve precise drug delivery [73, 74]. Therefore, accurately predicting the antibody–protein binding characteristics is crucial for understanding the immune response mechanism and developing new antibody drugs. AF3 perfectly meets this demand. Studies have shown that AF3 can accurately predict the key structural characteristics of antibody–protein interactions. Its accuracy rate is 33.3% higher than that of AFM v2.3, and the prediction accuracy rate of protein–protein complexes has also increased by nearly 10%. These technical advantages make AF3 a powerful tool for target identification and verification, not only accelerating the structural resolution of known targets but also helping in the discovery of new potential drug targets.

During the drug optimization stage, the technical advantages of AF3 are prominent. As an important link in the research and development of new drugs, the key to drug optimization lies in balancing efficacy and safety. Due to the lack of precise prediction of molecular interactions, the tissue-specific delivery efficiency of targeted drugs is not satisfactory. In addition, drug molecules are susceptible to interference from various factors in a complex biological environment, often resulting in delivery route deviation, reduced efficacy, and unexpected toxicity. These bottleneck problems have severely restricted the efficiency and success rate of new drug development.

At present, molecular docking technology has become a common method to evaluate drug affinity and binding ability [75–79]. By simulating the binding process of candidate drug molecules and target proteins, it can provide an important basis for drug design. In the tests conducted by the DeepMind team, the full-atom docking accuracy of AF3 reached 76.4%, which was 1.8 times that of RoseTTAFold All-Atom (RFAA) [80] docking (42%), and was also superior to other classic docking tools, such as Vina [81, 82], Gold, and Uni-Mol docking V2. It is particularly worthwhile to mention that AF3 performs exceptionally well in predicting covalent modifications (including ligands, glycosylation, and protein residue modifications, etc.), with the accuracy rates of all predictions exceeding 40%. This characteristic gives it a unique benefit in the development of targeted drugs based on covalent binding. Taking advantage of this, scientists not only precisely locate the binding sites of ligands and receptors [83], but also make preliminary judgments on the effects after drug modification through their predictions [84].

With these characteristics, AF3 is widely used in the research and development of drugs for various diseases. In neurological diseases, AF3 successfully conducted high-resolution modeling of over 1 200 brain-related proteins [with an average Predicted Local Distance Difference Test(pLDDT) > 80] and identified multiple potential drug targets related to Alzheimer's disease [85]. By integrating the Mendelian randomization method, the MR-SPI (a new MR method) identified seven proteins that underwent structural changes due to missense mutations (such as TREM2, CD33, etc.), providing new clues for the etiological study of Alzheimer's disease [86]. Furthermore, AF3 predicted the interaction between the W-Tau peptide-derived from intron 12 of the tau protein and tau monomers. Experiments confirmed that this peptide could not only inhibit tau aggregation but also depolymerize the paired helical filaments in the brains of patients with Alzheimer's disease, demonstrating great therapeutic potential [87].

Similarly, in the field of cancer treatment, AF3 successfully predicted the strong interaction between fatty acid hydroxylase domain containing 2 (FAXDC2) and guanosine diphosphate (GDP), and experimentally confirmed the inhibitory function of GDP on liver cancer cells, providing a new idea for targeted therapy of liver cancer [88]. In addition, by combining it with tools including ProteinMPNN and RFdiffusion, researchers designed nanoantibodies targeting proliferating cell nuclear antigen (PCNA) and B-cell lymphoma 6 protein (BCL6). The bioPROTAC system successfully degraded the target proteins and activated p53, providing a new strategy for cancer treatment [89]. In addition, scientists used this model to study the function of the Kirsten Rat Sarcoma Viral Oncogene Homolog 4B (KRas4B) derived CaaX peptide and found that it could interfere with the Ras signaling pathway by inhibiting farnesyltransferase, providing a new targeting possibility for Ras mutation-induced cancers [90].

For virus research, the low cost and convenience of AF3 have been timely in solving the problem of virus mutations. The AF3-assisted MT-TopLap topological deep learning method can accurately predict the free energy changes of receptor binding domains and angiotensin-converting enzyme 2 (ACE2) mutations in different species, providing an effective tool for rapid response to viral mutations [91]. At a deeper level, combining molecular dynamics and free energy perturbation, the changes in the binding free energy of the SARS-CoV-2 main protease mutant and Nirmatrelvir were analyzed, which further revealed the drug resistance mechanism of the viral mutant and guided the optimization of a new generation of inhibitors [92]. Based on its structural prediction, AF3 is also used to explore the interaction between anti-HIV drugs and targets [93], and to screen out peptides with higher affinity for targets from the newly synthesized peptides, significantly reducing the risk of drug resistance [94]. In addition to HIV, researchers also used AF3 to predict the dimer structure of the I7L protease of the monkeypox virus and the dynamic capsid conformation near the active site. This revealed the structural basis of substrate recognition to guide the design of covalent peptide mimic inhibitors with nanomolar efficacy [95].

In addition to the examples mentioned above, AF3 also plays an important role in drug development for diseases, such as fatty liver [96], muscle atrophy [97], and diabetic cardiomyopathy [98], including animal drugs [99]. The promotion of these applications all emphasize the key value of this model in the field of drug design. With the continuous updating and improvement of the open-source version, AF3 is bound to drive drug research and development towards shorter cycles, better efficacy, and fewer side effects, providing strong support for addressing global health challenges.

### Biomedical field

With the continuous development of cutting-edge technologies, such as big data and AI, the biomedical field is undergoing an unprecedented change. Driven by this wave, we have gradually entered the era of precision medicine. In order to achieve better therapeutic effects, researchers have been exploring the structure of biological macromolecules for decades. To obtain accurate structural information efficiently is the goal that this field has been pursuing. However, it is clear that traditional experimental methods cannot meet the growing demand and scientists have begun to turn their attention to the field of AI. Deep learning models, such as AlphaFold, ESMFold [100], and RoseTTAFold [101], have gradually become common tools for structure prediction in recent years, permitting the rapid development of the biomedical field. However, the limitations of their prediction accuracy and range have always been difficult problems to solve. Against this backdrop, the launch of AF3 is undoubtedly exciting and its high accuracy and wide applicability open up new opportunities in various areas, such as mechanistic research, vaccine development, protein engineering, and precision medicine.

#### Research on disease mechanisms

Study of the mechanisms of related diseases is an indispensable part of the biomedical field and serves as the foundation for drug development and disease treatment. The fundamental approach is to reveal the underlying causes through the disease manifestations, explore possible related signaling pathways and interactions, and provide theoretical support for the prediction, intervention, and prognosis of the disease. The conformation, modification, and interaction with other biomolecules of proteins are crucial for the study of life activities and disease mechanisms. Therefore, AF3 has been widely applied in this field. When exploring potential therapeutic targets for calcifying aortic valve disease, researchers used AF3 to simulate the interaction between two factors and combined it with other methods to explore how these factors inhibit valve calcification, lending a new targeted solution for the treatment of this disease [102]. In addition to predicting the connection between two biomolecules, AF3 can also predict diseases caused by conformational changes of proteins. In research of small round cell sarcoma, by combining other algorithms, AF3 constructs models of key proteins in the disease state and compares them with the wild type. While revealing the conformational changes, it analyzes the stability and affinity of the proteins, thereby assisting researchers in discovering the pathogenesis and potential breakthroughs of the disease [103]. AF3 is also very popular in basic mechanism research. Some researchers take advantage of its properties to explore a wide range of binding objects of a certain protein and search for potential targets of action [104]. Others also explore the mechanism of action of the target protein through its prediction of protein–protein interactions [105]. Some others have also demonstrated a new protein structure through its simulation function [106]. All these applications have demonstrated the significance of AF3 in mechanism research. It will become an excellent auxiliary tool to help people present the microscopic world of proteins.

#### Vaccine development and optimization

The principle of vaccine action is to activate the corresponding antigen–antibody response in an artificial way to achieve the rapid response of the body's immune system when the pathogen is invading. Because of its excellent performance in antigen–antibody docking prediction, AF3 has a pivotal role in the field of vaccine development. The core of a vaccine is the antigenic part that stimulates the body to produce an immune response. The model prediction is promising to allow researchers to quickly understand its antigenic structure, accelerating the preparation and optimization of vaccines. This effect has been implemented in AF2. By taking advantage of the structural prediction function of AF2, researchers have developed a hemagglutinin stem cell vaccine [107], which is a major breakthrough in the prevention and control of influenza B. By combining the reverse vaccinology method, researchers successfully designed a peptide-based vaccine against human respiratory syncytial virus using AF3 and verified its structural stability, which is crucial for the efficacy and safety of the vaccine [108]. These achievements attest to the feasibility of using deep learning models for vaccine design and provide more options for subsequent vaccine research and development.

#### Protein engineering

As a new research field, protein engineering has been improving rapidly with the development of the era. It mainly achieves the change of protein structure by artificial means, and even creates new proteins. In this field, how to accurately screen the desired proteins and how to efficiently achieve the directed evolution of proteins are invariable problems. At the World Conference on Artificial Intelligence in 2024, the launch of NewOrigin indicates that AI also has significant advantages in protein engineering. Coincidentally, EVOLVEpro, developed by the Massachusetts General Hospital Brigham in the United States, effectively achieved the directed evolution of proteins, as has been demonstrated in six proteins [109]. In a recent study, AF3 guided the engineering of the enNlovFz2 system [110]. This is a miniature eukaryotic gene editing tool. After engineering, both its recognition range and editing efficiency have been significantly improved. In addition, through the simulation of hydrophobic and hydrophilic structures of proteins, AF3 can provide guidance for the engineering of water-soluble integrated membrane proteins, which is of great significance for subsequent drug design and disease treatment [111, 112]. These achievements all reflect the revolutionary changes AI has brought to the field of protein engineering. We have reason to believe that AF3, as one of the best developments, will certainly bring greater surprises to the field in the future.

#### Precision medicine

Precision medicine is a major trend in modern medicine. It specifically refers to a standardized targeted treatment plan tailored for the alterations of biomarkers, such as genes and proteins, in a particular patient group. Focusing the target on specific molecular mechanisms can not only ensure the therapeutic effect but also reduce damage to the body during the treatment. Genomics, transcriptomics, and proteomics are some of the important analysis methods of precision therapy, and AI plays an important role in these endeavors. In 2023, an article published in Science detailed the use of whole-genome sequencing and deep learning tools to analyze variations in primate genomes to detect the pathogenicity of their variants [113]. Before that, the related structure sets of neurodevelopmental disorders (NDD) and uveal melanoma were also sorted and summarized through AlphaFold prediction and wet lab experiments [114, 115]. Based on these studies, AF3 was broadly applied in the field of precision medicine as soon as it was released. Due to its accuracy and wide applicability, this model has the advantage over other deep learning tools and can accurately simulate the structural and energy differences between the wild type and mutants, intuitively reflecting the influence of gene mutations on protein conformation and molecular interactions that is not limited to proteins. For instance, AF3 may dissect complex disease mechanisms of an aggressive type of Duchenne muscular dystrophy (DMD) by identifying the change in binding structure of a compound genetic variant in the DMD and TNNI3K genes, potentially imrpoving clinical management. [116]. In genetic hereditary diseases, the advantages of AF3 are more prominent. Neurofibromatosis type 1 caused by mutations in the NF1 gene has always been a difficult problem in the medical field. To visually discover the changes in protein conformation caused by each mutation site, researchers simulated the mutant protein through AF3, revealing the key impact of the R1000C mutation on the disease [117]. In addition, AF3 has been widely applied in the research of brain development defects [118], Marfan syndrome [119], fetal adipose granulomatosis [120], intellectual development disorders [121], and other diseases. These achievements provide good evidence of how precision medicine can promote the development of modern medicine.

### Other application fields

In addition to the above mentioned areas, AF3 also displays extensive potential in many other fields, including botany, nanomaterials, and bacterial research. These potential capabilities take AF3 to the next level.

#### Botanical research

Long before AF3 appeared, deep learning models were widely used in the field of plant immunity. These advanced algorithms chiefly provide brand-new technical means for plant science research by predicting protein structures and protein interaction networks. For plant disease prevention and control, researchers precisely simulated the interaction between pathogenic microorganisms and host plants, and screened out potential antiviral targets from complex molecular networks, providing a theoretical basis for the precise prevention and control of crop diseases [122]. Further research indicates that by conducting in-depth analysis of the protein structure of pathogenic bacteria carried by crop pests, the molecular mechanism of the interaction between viruses and their hosts can be revealed, benefiting the integrated management of pests. In addition to dealing with external biological stress, deep learning models also have significant value in the study of the intrinsic functions of plants. By analyzing the 3D structure of the protein encoded by key functional genes, researchers can intuitively understand its molecular mechanism, which provides an important reference for molecular design breeding to enhance crop disease resistance and increase yield [123].

With the release of AF3, botany research has been provided with more powerful tools. A recent study explores in detail how AF3 can be used to distinguish between sensor and helper proteins in plant nucleotide binding and leucine-rich repeat (NLR) immune receptors [124]. This achievement allows researchers to identify new disease resistance genes more quickly and accurately, and provides a new research idea for crop disease resistance breeding. In the field of agricultural pest control, researchers have successfully analyzed the structural characteristics of the flavivirus carried by the cabbage whitefly (*Aleyrodes proletella*) using AF3, providing an important basis for the development of new biological control methods and strongly promoting the sustainable development of agriculture [125]. In plant genomics, AF3 predicted the protein structure expressed by the GRAS gene family, verifying its conservation and functional diversity, and laying a theoretical foundation for the genetic improvement of important crops like sugarcane [126]. Similar research methods have also achieved important breakthroughs in rice breeding. Through the structural prediction of AF3, researchers have confirmed the specific interaction between plant A/T sequences and zinc-binding proteins (PLATZ) and DNA, clarifying their identity as transcription factors [127]. As PLATZ plays a key regulatory role in the growth and development of rice, this discovery provides a new molecular target for the improvement of rice varieties. The wide application of AF3 in botanical research not only deepens our understanding of plant immune mechanisms, but also injects new vitality into the sustainable development of agricultural production. From basic research to applied development, AF3 is driving botanical research to new heights.

#### Nanomaterials

Long before the rise of AF3, AI had already taken its place in the field of nanotechnology. By leveraging its database and high-throughput screening, researchers have significantly reduced experimental costs and achieved higher synthesis efficiency, which has been widely applied in the design, synthesis, and practical application of nanomaterials [128]. As a rising star in AI, the potential of AF3 in this field has also been rapidly tapped. Researchers used the AF3 AI model to assist the design of DNA nanostructures, and successfully constructed and optimized self-assembled triangular DNA structures [129]. This study not only demonstrates the application potential of AF3 in the field of DNA nanotechnology, but also provides new design ideas and strategies for more complex biomolecular self-assembly in the future. This discovery is expected to promote the development of nanotechnology and have a considerable positive impact on biomedicine, materials science, and other fields.

#### Bacterial research

With its outstanding prediction accuracy and wide application range, AF3 is triggering a revolution in the field of bacterial research. Compared to traditional experimental methods, AF3 can complete high-precision structural predictions of bacterial proteins and their complexes within a few hours, which provides unprecedented opportunities for understanding the physiological and pathogenic mechanisms of bacteria and developing new antibacterial drugs. In the research of bacterial adhesion mechanisms, AF3 successfully resolved the 3D structure of *Aeromonas hydrophila* repeats-in-toxin (RTX) adhesin and accurately predicted its three ligand-binding domains, including a carbohydrate binding module that can specifically recognize fucosylated glycans, providing a structural basis for the development of anti-adhesion inhibitors based on sugar analogues [130]. In the field of metabolic regulation, AF3 has been used to predict the structural characteristics of enzymes related to glycogen metabolism in methanogens, revealing the unique energy storage and utilization mechanisms of these archaea [131]. Furthermore, AF3 also contributed to the analysis of the dynamic conformational changes of CpaF ATPase during the assembly of Tad fimbria, clarifying the molecular mechanism that drives fimbria assembly through a rotational mechanism [132]. In the research of pathogenic microorganisms, AF3 not only successfully predicted the 3D model of the rib structural protein of *Trichomonas fetuses* [133], but also revealed the homologous domain characteristics of the cryptococcal sexual reproduction regulatory protein Sxi1α and its regulatory role in cell fusion [134]. It is particularly worth mentioning that through a systematic analysis of the functional domains of *Acinetobacter baumannii* phage caudin/spike protein, AF3 helped identify 32 key functional domains, providing important target information for the development of novel phage therapies [135]. These breakthroughs fully demonstrate the strong potential of AF3 in fundamental research and application development of treatments for bacterial infection.

## Potential obstacles in AF3 applications

An outstanding example of deep learning in biomolecular structure prediction, AF3 demonstrates its ability to simulate chemically diverse entities. The diversity of its functions undoubtedly broadens its application scope. However, due to the limitations of the techniques and methods, the complexity and variability of the structure, and the limitations of training and databases, AF3 has not brought about the disruptive innovation of structural forecasting expected. Some of the limitations inherent in deep learning models are also present in AF3 and remain the main challenges in this field.

### Lack of dynamic information

AF3 is reported to be able to provide highly accurate predictions for almost all existing complexes of molecular types that have been uploaded to the PDB. However, this achievement also reveals AF3’s reliance on existing crystal structures and cryo-electron microscopy data as training sets [73]. As such, its prediction results are essentially still “molecular photos” and cannot capture the dynamic conformational changes of the biomolecular system in the solution. Even by diffusion or using multiple random seeds, it is difficult to obtain an approximate solution set. Understanding the molecular mechanisms in the organism and cells often requires more intuitive dynamic processes. In studying other dynamic processes like metabolism, although the protein structure predicted by AF3 provides site information, it cannot simulate the conformational dynamic changes triggered by energy charges. This limitation restricts the application of AF3 in the study of biomolecular dynamic processes and drug dynamic interactions [136].

### Deviation of conformational state

Consistent with other deep learning tools, AF3 may provide incorrect or incomplete predictions of protein structural conformation. The original article describes this situation by taking E3 ubiquitin ligase as an example. In the real case, the enzyme is open under apo (no ligands) conditions, while it exhibits a closed conformation under holographic conditions (ligand-binding) [137]. However, predictions from AF3 showed that E3 ubiquitin ligases were in a closed conformation with or without ligands. Similarly, researchers also investigated the predictive effects of RFAA [80] and AF3 for protein–ligand interactions [138]. The results noted that when subjected to biologically credible perturbations, the predictions of either tool differ significantly from the expected physical behavior. In addition, predictive studies on G protein-coupled receptors (GPCRs) showed that although the performance of AF3 was improved compared with AF2, it could not achieve the effect of traditional methods in predicting ligand status [139]. These phenomena suggest that AF3 is not completely reliable in predicting intrinsically flexible regions or domains in complex structures. Due to the high standard of protein structure required in drug design, this means that AF3 cannot currently achieve the desired effect of independently supporting drug design and development. Therefore, how to achieve the polymorphism prediction of protein structure by AI is still an urgent problem for researchers to solve [71].

### Limitation of prediction accuracy

In the field of structure prediction, the prediction accuracy of AF3 has made significant progress, but there is still some room for improvement, especially for specific targets, such as orphan proteins. Due to the lack of known sequence-based near-origin homologues, AF3’s prediction results for this class of proteins are often unsatisfactory. In addition, the accuracy of AF3 in protein monomer structure prediction has not been significantly improved. This indicates that the AlphaFold platform needs to be further improved in the field of pure protein structure prediction.

For the field of drug development and vaccine preparation, prediction accuracy is an important index to measure the reliability of prediction results. When the prediction accuracy is <80%, whether it can meet the requirements of pre-screening becomes a problem worth pondering. Especially for those proteins with complex structures and prediction accuracy close to 50% or less, the reliability of the predicted results needs to be carefully evaluated. In addition, the insufficient content of the dataset is one of the important factors affecting the reliability of AF3 prediction results. This may cause AF3 to lack sufficient information support in the forecasting process, thus impeding the accuracy of its predictions. Although this problem can be mitigated to some extent by extensive prediction, the resulting computational costs are undoubtedly a challenge for researchers with limited resources.

Similarly, while AF3 is good at predicting protein interactions, it still has some potential limitations when dealing with highly flexible protein regions. The large conformational fluctuations in these regions can easily lead to deviations in the prediction results. Studies have shown that although AF3 has high accuracy in protein–protein complex prediction, it still has shortcomings in processing specific regions [38]. Although AF3 has improved antibody–antigen interaction prediction compared to the previous model (AlphaFold2-Multimer), the overall prediction accuracy still failed to meet the requirements of practical applications [140]. These defects have also been found in the prediction of protein–ligand [139, 141] and protein–nucleic acid interactions [142, 143].

Similar problems exist in the field of RNA structure prediction. Multiple systematic evaluations have shown that for long-chain RNA molecules, the model predicted by AF3 often has structural integrity problems, and the molecular shape parameters are significantly different from the experimental observations [144]. More crucially, due to the difficulty of AF3 has in capturing the inherent conformational heterogeneity of RNA molecules and the scale limitations of existing RNA structure databases, the prediction results seem inconsistent with the observed results [145]. In comparative tests of professional RNA structure prediction tools, the overall performance of AF3 was significantly inferior to those prediction methods specifically optimized for RNA characteristics, with deficiencies in the prediction of long sequences and non-classical interactions [146]. These limitations have, to some extent, hindered the wide application of AF3 in the field of drug development and biomedical research.

### Occurrence of hallucination effect

In the updated iteration of the model architecture, AF3 showed some new error types that did not occur in its predecessor AF2. When dealing with disordered regions of the protein, AF3 sometimes generates a false structural order, which is known as “hallucination” [147]. The occurrence of this phenomenon mainly stems from the model's incorrect prediction of the structure under the condition of insufficient information. The essence of the diffusion model is to obtain the predicted atomic coordinates through autonomous iterative denoising after learning the noise pattern through training. Using the existing dataset, the disordered noise gradually forms a clear structure. However, since biomolecules themselves may have disordered regions, AF3 can denoise this part as disordered noise during the prediction process, ultimately resulting in a false ordered structure. This characteristic will interfere with the researchers' correct judgment of molecular structure. Hallucination regions are often accompanied by extremely low confidence levels, with pLDDT scores well below the 50 threshold. Since these hallucinatory regions lack the distinctive banded appearance features that AF2 produces, such as the formation of alpha helical structures, researchers may misjudge protein structure prediction using AF3, especially in the prediction of phosphorylation modification regions [148].

**Figure 4.**
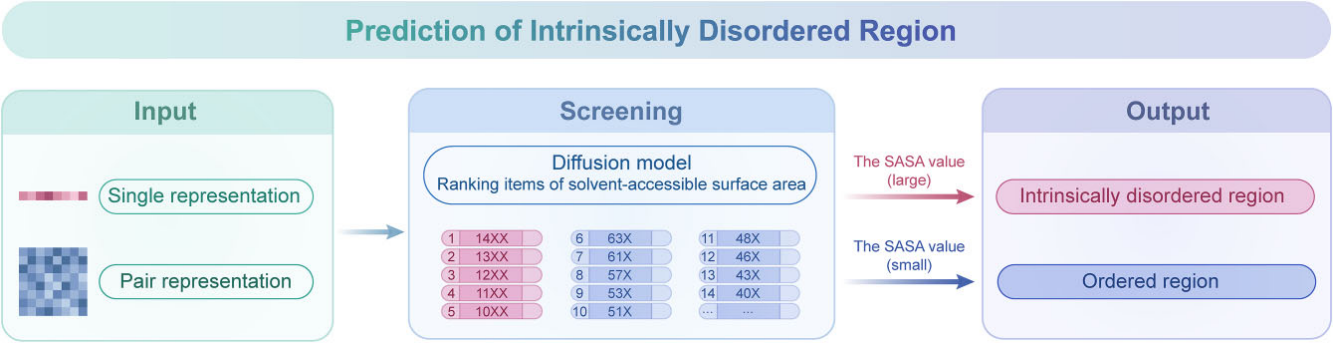
Schematic diagram of intrinsically disordered region prediction. The model adopts two input methods, namely single representation and pair representation, and generates protein conformations through the diffusion model. The prediction results were screened through the SASA ranking items, where the values (such as 14XX, 11XX, 63X, etc.) represent the SASA scores of different residues or fragments, referring to the solvent-exposure degrees of different conformations. The higher the value, the better the solvent accessibility and the more likely to be a disordered region. This approach might help distinguish the intrinsically disordered region and reduce the occurrence of hallucinations.

To address this challenge, AF3’s developers adopted a strategy of cross-distillation and encouraged the formation of structures with a larger solvent-accessible surface area (SASA) by introducing a ranking item (Fig. 4). The SASA refers to the area of the protein surface exposed to the solvent. Under normal circumstances, the SASA of disordered protein structures is larger than that of ordered structures. Therefore, the introduction of ranking items limits the conformation predicted by the model to a certain extent, making it closer to the natural structure of the protein. Nevertheless, the hallucination phenomenon is still difficult to completely eliminate and, to a certain extent, it has a negative impact on the predicted results of AF3. Therefore, how to further reduce the occurrence of hallucination and improve the accuracy of AF3 structure prediction in disordered regions is still an important direction of future research.

### Errors of stereochemistry

AF3 is also not perfect in stereochemistry. The prediction accuracy of AF3 chiral structure is insufficient, especially for predicting the conformation of small molecules. Although AF3 incorporates a penalty mechanism for chiral violations into its algorithm design, it has a chiral violation rate of 4.4% in the PoseBusters benchmark. This significant defect may adversely affect the analysis of protein structure and function and the screening of targets, thereby limiting the potential of AF3 applications in related fields. For example, in the conformational prediction of fatty acids, the detailed structure and interaction of AF3 generation are contrary to the experimental results [141]. In addition, AF3 occasionally has atomic overlap (conflict) during the prediction process [144]. This conflict sometimes manifests itself in extremes. That is, in a protein structure with two or more identical chains (homologues), the entire chain is incorrectly predicted to be in an overlapping state. Although AF3 reduces the occurrence of such phenomena to some extent by introducing a strategy of punishing conflicts, unfortunately the strategy does not completely eliminate the problem of atomic overlap.

In summary, while AF3 is an important advance in the field of protein structure prediction, like all scientific tools, it is undoubtedly not perfect. In a recent prediction test for CASP16, AF3 did not rank first as expected. Although it has a good predictive effect, it is inevitably inferior to the top-ranked teams when facing some complex tasks. This result shows that AF3 is not the only seed player in the protein prediction racetrack, and other prediction models may have their own areas of excellence. These tools need to be applied with caution and wisdom.

## Prospects

At present, AI and computer technology are gradually penetrating the field of scientific research, and the pharmaceutical and biomedical industries are experiencing an unprecedented renaissance. As a currently emerging structure-prediction model, although AF3 itself still has many insurmountable defects, with continuous optimization after open source collaboration, a comprehensive analysis of protein structure has already seen light at the end of the tunnel [48], and its application prospects in the fields of drug research and development and biomedicine show a multi-dimensional development trend.

Judging from the current situation, overcoming the inherent defects of AF3 requires the joint efforts of other tools [70]. In previous studies, some researchers have found that combining AF3 with transfer learning techniques can overcome the model's dependence on the structure of natural proteins and MSA [149]. Furthermore, experiments have confirmed that template-based modeling outperforms AF3 in the prediction of ligand binding sites [150]. Therefore, the combination of AF3 with template-based modeling can optimize the identification of confused residues through hydrophobic MSA, significantly improving the accuracy of ligand binding-site prediction. In the process of protein complex stoichiometric prediction, the fusion of AF3 and homologous template information can increase the TOP1 accuracy rate to 71.4%, highlighting the advantages of multimodal methods in the resolution of quaternary structures [151].

These achievements demonstrate the feasibility of AF3 for performance optimization in combination with other methods. From this reasoning, seeking predictive tools (such as Disobind [152]) for internal disordered regions or other special structures and using them in combination with AF3 might be a good countermeasure to solve the hallucination effect. Existing studies have shown that introducing dynamic network analysis and molecular dynamics simulation into AFM can significantly improve the prediction accuracy of epitopes, especially in the prediction of complex conformations such as vertical binding modes [153]. This successful experience can be directly transferred to the optimization process of AF3. To improve the prediction accuracy of AF3 in heterogeneous RNA, a hybrid strategy combining calculation and experiments (such as atomic force microscopy) can be considered [145]. Through the structural constraint information provided by the experiment, the prediction results obtained by the pure calculation method are verified and optimized, thereby significantly improving the reliability of the prediction model. Furthermore, regarding the deficiencies of AF3 in the prediction of antigen–antibody interactions, the recently developed Cmai model [154] (a deep learning framework specifically designed for the prediction of antigen–antigen binding) and the epitope-paratope predictor (EPP) [155], can be used as effective supplementary tools to enhance prediction reliability in this field.

In addition to the performance optimization of the prediction model, the expansion of the training set is also a point that AF3 must pay attention to in its future development. Integrating AF3 with other datasets, such as transmembrane protein databases (e.g. TMVisDB [156]) and ion channel resources (e.g. PLIC [157]), can provide high-quality structural and functional correlation data for the iterative training of the model. Similarly, the construction of a PTM rotational isomer library can optimize the structural prediction of modification sites by AF3 [158], while the expansion of protein–small molecule drug databases (such as DrugDomain [159]) can enhance the modeling ability of AF3 for small molecule binding interfaces. With the emergence of open-source tools (such as af3cli [160]) and automated processes (such as MULTICOM4 [161]), the input generation and model evaluation of AF3 will be further simplified, greatly promoting the popularization of AF3.

Although AF3 has broad application potential, we must clearly realize that in practical application scenarios such as drug development, vaccine preparation, and so on, relying solely on AI prediction methods like AF3 is far from ideal or absolutely accurate. Developing a drug or therapeutic into the clinic requires a high degree of precision in protein structure, and even a small miscalculation can have serious consequences. The prediction accuracy of AF3 still cannot meet the requirements in the field of drug design, and as its prediction ability means that it cannot replace experimental results, traditional experimental methods still occupy an indispensable position [162]. Therefore, the advent of deep learning models such as AF3 is fundamentally an auxiliary tool to traditional methods, laying the necessary early foundation for drug development and biomedical fields. It complements traditional experimental methods and jointly promotes the development and progress of related fields [163].

Although there are still many issues with the current AF3, we believe that time will change everything. With the continuous progress of technology and the continuous efforts of researchers, AF3 and its likely future versions may eventually evolve into a perfect tool and play an increasingly important role in every field and immensely benefit the human being.
